# Standing Up from a Chair with an Asymmetrical Initial Foot Position Decreases Trunk and Masticatory Muscle Activities in Healthy Young Men

**DOI:** 10.3390/healthcare8040480

**Published:** 2020-11-12

**Authors:** Youngsook Bae

**Affiliations:** Department of Physical Therapy, College of Health Science, Gachon University, Incheon 21936, Korea; baeys@gachon.ac.kr; Tel.: +82-32-820-4324

**Keywords:** asymmetrical foot position, muscle activation, sit-to-stand, trunk kinematics

## Abstract

This study aimed to identify the activation of lower extremity, trunk, and masticatory muscle and trunk kinematics of the initial foot position during the sit-to-stand (STS) movement. Sixteen young men participated in this cross-sectional pilot study and performed STS using both symmetrical and asymmetrical foot positions. Activation of the tibialis anterior (TA), gastrocnemius lateral head (GA), rectus femoris (RF), biceps femoris (BF), rectus abdominis, erector spinae (ES), sternocleidomastoid (SCM), upper trapezius (UT), temporalis (TE), and masseter muscles in the dominant side was determined. For trunk kinematics, head and trunk velocities, front-back (For-Back) and mediolateral (Med-Lat) weight translation rates, and trunk inclination were measured. GA, TA, BF, and RF activation significantly increased, whereas ES, SCM, UT, and TE activation significantly decreased when using the asymmetrical foot position. Head velocity, For-Back, Med-Lat, and trunk inclination were also significantly decreased. In conclusion, the asymmetrical foot position increases muscle activation in the lower extremities and decreases trunk inclination. In addition, ES, UT, and TE muscle activity decreases at the initial asymmetrical foot position.

## 1. Introduction

The average person sits and gets up from their chair an average of 60 or more times a day [1]. The sit-to-stand (STS) movement, the ability to go from a sitting position to a standing position, is an important skill essential for mobility and is closely related to the ability to live an independent life [2]. The entire body and lower extremity segment movements must be efficiently controlled using coordinated muscle activity during STS [3].

Standing from a sitting position is a continuous movement from the feet to the head [4]. Forward head posture or excessive head and neck movements could affect the lower spine and lower extremities [5]; conversely, during activities performed in a closed kinematics chain, foot posture may lead to postural alterations in pelvic and spine posture [6,7]. Therefore, repetitive STS can affect not only the lower extremities but also the head posture and masticatory muscle. As the accumulation of micro-damage caused by repetitive movements in daily life can result in changes in the body system [8], repeated STS behavior in daily life may result in changes in the activity of the neck and masticatory muscle as well.

Most studies of STS movements have identified their effects on the inclination of the trunk [9], the pattern of muscle activity in the lower extremity muscles [10] and how it is affected by the foot position [2], and the movement of the joints [11]. The determinants of an STS strategy are speed, foot position, trunk position, arm movement, and knee position [12], but particularly foot position. In the trunk aspect, range of motion, maximum angular velocity, and vertical velocity might be used to identify STS strategies [13]. One of the most important factors contributing to the success of standing from a chair is the muscle strength of the lower extremities of the user [14]. Standing from a sitting position with your feet in front of your knees [10] or excessive forward flexion of the trunk not only results in mechanical back pain [15,16], but can also result in movement disorders [17]. Performing STS in an asymmetrical position with one foot ahead of the planted leg increases the displacement and velocity of the trunk; as a result, the mass of the body moves forward [10]. Therefore, previous studies have suggested that performing STS in an asymmetrical position with one foot ahead of the planted leg is not a healthy posture. Alternatively, when one foot is positioned behind the planted leg, the ankle plantar flexion and knee extension moments increase during STS [2]. There have been studies on the muscles of the lower extremities during STS in an asymmetrical foot posture; however, studies on trunk posture and trunk and masticatory muscle activity are lacking, and the effects of repetitive STS on the trunk and masticatory muscle activities are not clear.

Therefore, this study evaluated how foot placement affects the back, neck, and masticatory muscle during STS movement. To directly assess this, lower extremity, trunk, and masticatory muscle activation was measured as well as trunk posture. These data were collected during STS movements using symmetrical and asymmetrical foot positions. We hypothesized that the activity of the lower limb muscles would increase during STS using the asymmetrical foot position, in accordance with the findings of previous studies. In addition, we predicted decreased trunk and masticatory muscle activity in addition to reduced trunk forward inclination and vertical speed.

## 2. Materials and Methods

### 2.1. Participants and Study Procedures

Sixteen healthy young men participated in this study. All participants met the following criteria: (1) had no history of either musculoskeletal or orthopedic disease, (2) could perform STS independently, and (3) had a body mass index of <30 because obesity limits the anterior spinal flexion during STS [18]. Surface electromyography revealed significant differences at all force levels of contraction in gender [19]. Therefore, only men were recruited in this study. Individuals who had previous balance, neurologic, or vestibular impairments or any contraindications to the measurement procedures were excluded. The participants were instructed not to engage in vigorous physical activity for 24–48 h prior to the study and were recruited using convenience sampling. The participant’s dominant and non-dominant foot were determined prior to the initiation of the experiments. The foot used to kick the ball was set as the dominant foot. The order of execution of symmetrical and asymmetrical foot strategies was randomized. All participants were informed in detail about the study procedure and safety precautions, provided written informed consent, and trained to become familiar with how to perform STS prior to the study.

The experiment was conducted according to the latest version of the Declaration of Helsinki. All procedures of this study were approved the Human Ethical Review Committee (201503-HSR-027-1). All data collection was undertaken at a university laboratory. Outcome assessors and data analysts were blinded to the study purpose. This study used the G*Power software (Version 3.1.9.4, Kiel University, Kiel, Germany) to calculate the appropriate sample size. Using a power of 80% and a significance level of *p* < 0.05, a sample size of 16 participants was calculated to be necessary.

### 2.2. STS Procedure

To perform the STS procedure, participants sat upright on a height-adjustable table without a backrest or armrest. The arms were placed in a relaxed position parallel to the trunk. Each participant’s seat height was set in level with the center of the knee joint (patellar) and feet were placed shoulder-width apart. During the STS procedure, participants sat upright with their hand next to the trunk. Then, participants performed STS on the verbal cue of “start.” In the symmetrical foot position, the knees are bent 90° and the feet are placed on the floor with both heels directly below the knees. In the asymmetrical foot position, the toes of the dominant foot were placed behind the heel of the non-dominant foot [2]. Subjects performed sequential trials of each strategy, and the order in which the procedure was conducted first was randomized. The participants practiced 3 times before the study to be able to perform the STS procedure. After the practice, and completing the STS procedure, they stood for 5 s; the maximum time until STS was performed after sitting on the chair was 1 min. Participants were measured three times for each foot position and the mean was scored.

### 2.3. Measurements

#### 2.3.1. Muscle Activation

A wireless telemetry Electromyography (EMG) system (Telemyo 2400GT, Noraxon USA, Scottsdale, AZ, USA) was used to determine the EMG signals. Adhesive pre-gelled Ag/AgCl surface EMG electrodes (Delsys Inc., Boston, MA, USA) were placed on six trunk and lower limb muscles (rectus abdominis (RA), erector spinae (ES), rectus femoris (RF), biceps femoris (BF), tibialis anterior (TA), and gastrocnemius lateral head (GA)) and four additional muscles (temporalis (TE), masseter (MA), sternocleidomastoid (SCM), and upper trapezius (UT)), all on the dominant side. The positioning of the electrodes was as described previously [20,21,22].

After attaching the electrodes, participants performed maximum voluntary isometric contractions (MVIC) of each muscle in the manual muscle test posture, and average values were calculated from three distinct measurements. In the supine position with 90° knee flexion, RA and SCM were measured. For RA, participants put their hands behind their heads and flexed the trunk 45°. Resistance was then applied to the chest. For SCM, participants turned toward their non-dominant side and lifted their head off the table while resistance was applied to the forehead. For ES, patients started in the prone position with their fingertips touching the side of their head and their chest off the table; resistance was applied to the scapular. For BF, patients laid in the prone position with their knee pointed laterally and flexed to 45°, with resistance then applied on the ankle. RF, TA, UT, MA, and TE were all measured while being seated on a chair (hip flexion 90° and knee flexion 90°). The MVIC of RF was measured while straightening the knee and applying resistance to the ankle. For TA, the foot was brought up and in (toward the body), providing resistance on the medial aspect of the foot over the first toe. The MIVC of UT was measured by elevating the shoulder while applying resistance to the shoulder in a downward direction. To measure MA and TE, a tongue depressor was placed in patients’ molars and patients were asked to chew for as long as they could. For GC, participants stood on the dominant foot to be tested with their knee extended and were asked to raise their heel from the floor as high as possible.

The EMG sampling signal was set at 1500 Hz and the obtained raw data were subjected to full-wave rectification. The rectified EMG values were smoothed again, (RMS: 100 ms) and the band pass filter was set at 20–500 Hz, with the notch filter set at 60 and 120 Hz. EMG signals were standardized to EMG signals measured during MVIC before STS.

#### 2.3.2. Trunk Kinematics

After MVIC measurements, the following trunk kinematic parameters were measured using the DIERS formetric 4D analysis system (DIERS International GmbH, Schlangenbad, Germany): head velocity, trunk velocity, front-back (For-Back) weight translation rate, mediolateral (Med-Lat) weight translation rate, and trunk inclination. The DIERS formetric 4D analysis system allows rapid static and dynamic optical measurement of the human back and spine. The procedure is radiation-free and operates without contact, capturing 50 picture frames per second for 5 s and converting these images into a 3D map of the spine. Spinal dynamics are measured in each frame, creating a real-time representation of the spinal movement as it occurs during STS. Head velocity and trunk velocity are defined as the speed at which the center of the head and trunk move vertically during STS. For-Back is defined as the rate of change in the center of mass when the body weight moves back and forth during STS, whereas Med-Lat is the rate of change in the center of mass when the body weight moves side to side during STS. Trunk inclination was measured from the line of gravity and the 7th spinous process of the cervical spine to the line connecting the centerline of the posterior superior iliac spine; the larger the number, the more the trunk is inclined forward.

### 2.4. Statistical Analyses

All statistical analyses were conducted using SPSS version 24.0 (IBM Corp., Armonk, NY, USA). Frequency analysis and descriptive statistics were used to describe the participants’ general characteristics. The comparisons of muscle activation and kinematics of the two STS strategies were investigated using the dependent *t*-test. All variables are expressed as mean ± SD. The threshold for statistical significance was set at *p* < 0.05. The significance level was set at *p* < 0.05.

## 3. Results

The average age of the participants was 23.63 years. The descriptive characteristics of the individuals are shown in Table 1.

### 3.1. Comparison of Muscle Activation between Symmetrical and Asymmetrical Foot Strategies

In STS, activation of the lower extremity muscles GA, TA, BF, and RF was more significantly increased in the asymmetrical foot strategy than in the symmetrical foot strategy (*p* < 0.01). Conversely, EC muscle activation as well as SCM (*p* < 0.01), UT (*p* < 0.05), and TE (*p* < 0.01) activation were significantly decreased in the asymmetrical foot strategy (*p* < 0.01) (Table 2).

### 3.2. Comparison of the Kinematics between Symmetrical and Asymmetrical Foot Strategies

Head velocity decreased significantly during STS using the asymmetrical foot strategy (*p* < 0.01). The rate of change in For-Back (*p* < 0.01) and Med-Lat (*p* < 0.05) weight translation rate also significantly decreased. Trunk inclination was also significantly reduced during the asymmetrical foot strategy (*p* < 0.01, Table 3).

## 4. Discussion

In this study, the activity of the lower extremity muscles increased during the asymmetrical foot strategy, whereas EC muscle activation and trunk inclination decreased, in addition to upper trapezius (UT) and temporalis muscle activation.

STS is considered the most mechanical movement in daily living activities due to the high level of muscle activation [23]; the individual must coordinate a transition from a horizontal to a vertical position in one complex movement [24]. Particularly in the lower limbs, muscle activation ensures greater autonomy in all activities performed during daily life [25]. STS is an anti-gravity movement; therefore, it needs to dynamically align various body segments and synergize the activation of many muscles, with the lower extremity muscles being very highly activated. In this study, STS was performed with the dominant leg positioned behind the non-dominant leg, and significant increases in the muscle activation of GA, TA, BF, and RF were observed. These results are similar to the results of a previous study that demonstrated an increase in ankle plantar flexion and knee extension torque of the posterior leg in an asymmetrical posture during STS [2].

In addition, increasing the forward trunk lean while running can be used as a strategy to reduce knee load [26]. Forward trunk lean is also associated with lower knee extensor moments during weight bearing [27,28]. During a healthy individual’s torso flexion, the increase in actual spinal load comprises the theoretical spinal load and the load produced by back muscle activity [29]. Older people with weak strength have been documented using different strategies to use the trunk more dynamically during STS [13], and sit-to-stand training combined with transcutaneous electrical stimulation may be used to improve the balance function and muscle strength [30]. As shown in Table 3, the activation of the lower limbs, trunk, neck, and temporomandibular joint muscles changed in the symmetrical and asymmetrical feet. Our results show that trunk inclination and paravertebral muscle activation decreased during STS with an asymmetrical foot strategy, whereas muscle activation of lower extremity muscles GA, TA, BF, and RF significantly increased. These findings indicate that the asymmetrical strategy with the dominant leg behind decreases the movement of the trunk and reduces ES muscle activation by increasing the activity of the lower extremity muscles during STS. In addition, prolonged trunk flexion increases the lumbar load [31]; when lumbar load is increased, back extensor muscle activation is increased, rather than abdominal muscle activation [29]. Therefore, as our asymmetrical foot strategy decreases ES activation, the lumbar load may also be reduced.

In the present study, the head velocity and For-Back and Med-Lat weight translation rates were reduced in the asymmetrical foot position. This is similar to the increased body stability after STS in an asymmetrical foot position [32]. This indicates that an asymmetrical posture with the dominant foot behind increases the activity of the muscles of the lower extremity, generating sufficient force and increasing the stability. In addition, it is believed that a decrease in the For-Back and Med-Lat weight translation rates increases trunk stability during STS. The authors suggested that the asymmetrical foot strategy increases the mechanical demands of the lower extremity muscles during STS, providing a desirable strategy to increase the muscle activity of the lower limbs and reduce ES muscle activation. In light of these data and our results, this asymmetric foot strategy during STS may serve as a posture for increasing lower extremity muscle activation during STS.

When the forward inclination of the trunk increases, the forward inclination of the head may also increase. This forward head inclination can change the resting position of the mandible and affect masticatory muscle activity [33,34], and this has been associated with increased activation of the temporal and masseter muscles [35] as well as changed UT muscle activation [36]. In the asymmetrical foot strategy, we found that trunk inclination decreased, along with temporalis, UT, and SCM muscle activation. Our findings suggest that the decrease in trunk inclination decreases the activity of the neck muscles and masticatory muscles when performing STS in an asymmetrical foot position. Considering previous work, an increase in temporalis muscle activation at rest should be recognized as an important indication of temporomandibular disorder [37]. We therefore speculate that repeatedly performing STS in the symmetrical foot position is an inappropriate posture for a person with temporomandibular disorder; nevertheless, further research is warranted to provide a clear basis for this.

Concerning limitations, this study compared the movement of the foot in an induced posture. This posture may suppress the normal and habitual posture adjustment of the head and neck. Furthermore, in accordance with a previous study [2], this study was performed only in one posture: with the dominant foot behind, thereby increasing muscle activity in the lower extremities. However, trunk and masticatory muscle activity in the opposite posture (with the dominant foot forward) was not measured. Additionally, the observed masticatory muscle activation was related to the movement of the neck; however, measuring the kinematics of the neck was beyond the scope of this study. To address these limitations, further research concerning the impact of the position of the foot during STS on trunk and masticatory muscle activation is warranted.

Despite these limitations, the study has several advantages. This study is the first to confirm trunk, neck, and masticatory muscle activation depending on the foot position in STS. Therefore, it provides the basis for further research to identify neck and masticatory muscle activity depending on the foot position in STS.

## 5. Conclusions

This study observed that STS with an asymmetrical foot position increases lower limb muscle activity and decreases trunk inclination and ES muscle activation. Decreased ES muscle activation relates to reduced lumbar load. Therefore, in STS, this asymmetrical strategy is also an appropriate posture to prevent lower back pain. Increased lower limb muscle activation also reduces head velocities and increases trunk stability. In addition, decreased trunk inclination reduces the forward head velocity, thereby reducing UT, SCM, and TE muscle activation. This indicates that our asymmetrical foot position reduces tension in the neck and masticatory muscles. Our findings are important for designing STS interventions for clinical populations, such as the frail, to improve muscle activity in the lower extremities and could suggest a suitable posture for performing STS to prevent back, neck, and Temporomandibular joint (TMJ) problems.

## Figures and Tables

**Table 1:** Baseline characteristics of the participants.

Characteristic	Total (*n* = 16)
Age (years)	23.63 ± 1.89
Height (cm)	174.69 ± 4.23
Weight (kg)	71.81 ± 6.30
BMI (kg/m^2^)	23.52 ± 1.88
Foot size (mm)	268.75 ± 7.18

**Table 2:** Comparison of muscle activation between symmetric and asymmetric foot strategy.

Variables	Symmetric Foot	Asymmetric Foot	Difference (95% CI)	t	*p*
Temporalis	3.27 ± 1.73	2.66 ± 1.34	−0.61 (−0.210~−1.013)	−3.253	0.005
Masseter	2.95 ± 1.45	2.51 ± 0.82	−0.43 (0.133~−1.013)	−1.635	0.123
SCM	7.44 ± 3.55	5.12 ± 2.31	−2.31 (−1.136~−3.490)	−4.190	0.001
Upper trapezius	32.33 ± 12.74	25.96 ± 8.05	−6.36 (−1.462~−11.265)	−2.768	0.014
Rectus abdominis	36.76 ± 19.66	31.23 ± 16.47	−5.02 (0.353~−10.398)	−1.991	0.065
Elector spinae	62.13 ± 18.33	49.24 ± 15.67	−12.89 (−9.331~−16.449)	−7.720	0.000
Rectus femoris	41.04 ± 15.90	67.20 ± 35.37	26.16 (42.293~10.029)	3.457	0.004
Bicpes femoris	30.82 ± 16.95	43.02 ± 20.75	12.19 (20.444~3.954)	3.154	0.007
Tibialis anterior	25.57 ± 9.31	50.94 ± 17.15	25.36 (32.243~18.493)	7.865	0.000
Gastrocnemius lat.	28.57 ± 18.37	40.22 ± 24.47	11.654 (16.460~!6.848)	5.169	0.000

SCM: sternocleidomastoid.

**Table 3:** Comparison of kinematics between symmetric and asymmetric foot strategy.

Variables	Symmetric Foot	Asymmetric Foot	Difference (95% CI)	t	*p*
Head velocity (m/sec)	2.09 ± 2.91	0.96 ± 0.10	−0.61 (−0.210~−1.013)	−3.253	0.005
Trunk velocity (deg/sec)	171.11 ± 82.59	166.07 ± 77.61	−0.43 (0.133~−1.013)	−1.635	0.123
For-Back (%)	49.49 ± 11.28	29.62 ± 7.71	−2.31 (−1.136~−3.490)	−4.190	0.001
Med-Lat (%)	29.30 ± 12.15	21.66 ± 7.34	−6.36 (−1.462~−11.265)	−2.768	0.014
Trunk inclination (degree)	33.25 ± 8.80	26.87 ± 8.27	−6.37 (−8.523~−4.226)	−6.326	0.000

For-Back: front-back weight translation rate, Med-lat: medio-lateral weight translation rate.
